# Effect of interpregnancy interval on the risk of gestational diabetes mellitus during a second pregnancy

**DOI:** 10.1186/s12884-024-06602-z

**Published:** 2024-06-04

**Authors:** Yuqing Deng, Chang Xu, Ao Yang, Ying Wang, Yanting Peng, Ying Zhou, Xiangzhi Luo, Yalin Wu, Shilin Zhong

**Affiliations:** 1 Center of Obstetrics and Gynecology, Peking University Shenzhen Hospital, 1120 Lianhua Road, Shenzhen, 518036 Guangdong China; 2 Institute of Obstetrics and Gynecology, Shenzhen PKU-HKUST Medical Center, Shenzhen, Guangdong China; 3 Shenzhen Key Laboratory on Technology for Early Diagnosis of Major Gynecologic Diseases, Shenzhen, Guangdong China; 4 Intelligent Hospital Research Academy, Peking University Shenzhen Hospital, Shenzhen, Guangdong China

**Keywords:** Gestational diabetes mellitus, Risk factor, Interpregnancy interval, Multivariate logistic regression, Maternal age

## Abstract

**Background:**

Interpregnancy interval (IPI) is associated with the risk of GDM in a second pregnancy. However, an optimal IPI is still need to be determined based on the characteristics of the population. This study aimed to analyze the effect of interpregnancy interval (IPI) on the risk of gestational diabetes mellitus (GDM) in the Chinese population.

**Methods:**

We conducted a retrospective cohort study on female participants who had consecutive deliveries at Peking University Shenzhen Hospital from 2013 to 2021. The IPI was categorized into 7 groups and included into the multivariate logistic regression model with other confound factors. Analysis was also stratified based on age of first pregnancy, BMI, and history of GDM. Adjusted OR values (aOR) and 95% confidence intervals (CI) calculated. The regression coefficient of IPI months on GDM prediction risk was analyzed using a linear regression model.

**Results:**

A total of 2,392 participants were enrolled. The IPI of the GDM group was significantly greater than that of the non-GDM group (*P* < 0.05). Compared with the 18–24 months IPI category, participants with longer IPIs (24–36 months, 36–48 months, 48–60 months, and ≥ 60 months) had a higher risk of GDM (aOR:1.585, 2.381, 2.488, and 2.565; 95% CI: 1.021–2.462, 1.489–3.809, 1.441–4.298, and 1.294–5.087, respectively). For participants aged < 30 years or ≥ 30 years or without GDM history, all longer IPIs (≥ 36 months) were all significantly associated with the GDM risk in the second pregnancy (*P* < 0.05), while any shorter IPIs (< 18 months) was not significantly associated with GDM risk (*P* > 0.05). For participants with GDM history, IPI 12–18 months, 24–36 months, 36–48 months, and ≥ 60 months were all significantly associated with the GDM risk (aOR: 2.619, 3.747, 4.356, and 5.373; 95% CI: 1.074–6.386, 1.652–8.499, 1.724–11.005, and 1.078–26.793, respectively), and the slope value of linear regression (0.5161) was significantly higher compared to participants without a history of GDM (0.1891) (*F* = 284.168, *P* < 0.001).

**Conclusions:**

Long IPI increases the risk of GDM in a second pregnancy, but this risk is independent of maternal age. The risk of developing GDM in a second pregnancy for women with GDM history is more significantly affected by IPI.

**Supplementary Information:**

The online version contains supplementary material available at 10.1186/s12884-024-06602-z.

## Background

Gestational diabetes mellitus (GDM) refers to diabetes diagnosed only during pregnancy. An estimated 1 in 6 live births worldwide is complicated by GDM [1]. The incidence of GDM in China is close to 14.8%, according to the International Association of Diabetes and Pregnancy Study Groups (IADPSG) criteria [2]. GDM has significant adverse effects on both the mother and fetus [3]. Pregnant women with GDM have a significantly increased incidence of metabolic diseases such as type 2 diabetes and obesity in the long term, and their offspring have a significantly increased risk of metabolic diseases including adiposity and diabetes when they grow up [4]. Risk factors for GDM include obesity, advanced maternal age, GDM in a previous pregnancy, and a family history of diabetes [5, 6].

Interpregnancy interval (IPI), which refers to the time between the end of a pregnancy and the start of another [7], is associated with the risk of poor perinatal outcomes. An excessively short IPI (less than 12 months) is widely believed to increase the risk of a variety of adverse pregnancy outcomes, such as uterine rupture [8], spontaneous preterm birth [9], and perinatal death [10]. A long IPI may be also associated with severe maternal morbidity [11]. The World Health Organization (WHO) recommends an appropriate IPI of at least 24 months [12], and the American College of Obstetricians and Gynecologists (ACOG) recommends an IPI of 18 months to 5 years [13]. The upper limit of the optimal IPI is still to be determined based on the specific population and disease state. However, studies on the association between a short IPI and the risk of GDM are contradictory. Studies from Canada [14] and the United States [15] have suggested that a short IPI increases the risk of GDM in the second pregnancy, whereas research from Australia found that a shorter IPI had a protective effect on the risk of GDM [16]. Results concerning the association between a long IPI and GDM also differ between studies. A study from China found that only an extremely long IPI of at least 120 months increased the risk of GDM [17], whereas data from the United States suggested that an IPI of over 36 months significantly increased the risk of GDM [15]. A large-sample study from Uruguay found that neither short nor long IPIs had a significant effect on the risk of GDM [18].

These different findings may be explained by differences in the study participants, confounding factors selected, and methods of analysis. With the adjustment of China’s birth policy, more women will be multiparous, and an appropriate IPI will be beneficial to prevent GDM in subsequent pregnancies. Therefore, we conducted this study to analyze the effect of IPI on the risk of GDM in the Chinese population to provide information for the prevention and treatment of GDM.

## Methods

### Subjects

We performed a retrospective cohort study of female participants with two consecutive single deliveries in Peking University Shenzhen Hospital over 9 years (2013–2021). The protocol for this study was approved by the Medical Ethics Committee of Peking University Shenzhen Hospital. The inclusion criteria for this study comprised women between the ages of 18 and 45 who were Chinese nationals and had experienced two consecutive pregnancies, both lasting a minimum of 28 weeks gestational age. Exclusion criteria for this study included women with a parity of one, women with a parity of three or more, women with unknown number of parity, women who delivered before 28 gestational weeks, women without available delivery date or gestational week of delivery, women with type 1 or type 2 diabetes, and women with unknown or unstated BMI for their first or second pregnancy. According to whether the second pregnancy was complicated by GDM, the participants were classified into the GDM group or the non-GDM group.

### Exposures

The IPI was defined as the months between the end of a pregnancy and the start of another. This was obtained by deducting the gestational months of the second pregnancy from the total months of the delivery interval between the two pregnancies. The IPI was categorized as follows: IPI < 12 months, 12 months ≤ IPI < 18 months, 18 months ≤ IPI < 24 months, 24 months ≤ IPI < 36 months, 36 months ≤ IPI < 48 months, 48 months ≤ IPI < 60 months, IPI ≥ 60 months. The category of “8 months ≤ IPI < 24 months” was selected as a reference when comparing the effects of different IPI categories on the risk of GDM in the second pregnancy.

The following data were collected from the delivery records of the hospital information system: IPI; maternal age; assisted reproductive technology (ART); parity; year of delivery; delivery mode; occupation; medical payment method; nationality; marital status; sex of newborn; birth weight in the first pregnancy; body mass index (BMI) of the first trimester in the first and second pregnancy; and whether GDM, thyroid disease, polycystic ovary syndrome (PCOS), hypertensive disorder complicating pregnancy (HDCP), or preterm birth occurred.

### Outcome

GDM in the second pregnancy was set as the outcome, which was diagnosed by a 75-gram oral glucose tolerance test using the IADPSG criteria [19]: blood glucose at 0 h of ≥ 5.1 mmol/L, blood glucose at 1 h of ≥ 10.0 mmol/L, or blood glucose at 2 h of ≥ 8.5mmol/L.

### Analysis methods

The IPI, age at the first pregnancy, birth weight of the newborn of the first pregnancy, BMI in the first and second pregnancy, and proportion of specified complications were compared between the two groups. Multivariate logistic regression was conducted to explore the independent risk factors for GDM in the second pregnancy. In addition, stratified analysis was conducted to analyze the effect of IPI on the GDM risk in specific populations divided by maternal age, BMI, and GDM in the first pregnancy. In stratified analysis, we determined the minimum sample size needed for each subgroup using an every per variable (EPV) of 10. We made this estimation by considering the proportion of GDM occurrence and the number of independent variables in the multivariate logistic regression model. If the initial stratification has a smaller sample size than the estimated minimum, we will modify the cut-off value of the stratification index to ensure that each subgroup fulfills the statistical criteria.

### Statistical analysis

SPSS 24.0 statistical software (IBM, Armonk, NY, USA) was used for the analysis of the data. Categorical variables were expressed as n (%) and tested by Chi-square test between groups. The normally distributed variables were expressed as mean ± standard deviation and tested by student’s *t*-test, whereas non-normally distributed variables were expressed as median (interquartile range; IQR) and tested using the Mann–Whitney *U* test. Multivariable logistic regression models were used to determine the association of IPI (independent variable) with the risk of GDM in the second pregnancy (dependent variable), adjusted for maternal age, BMI, PCOS, ART, GDM, HDCP, preterm birth, and cesarean section in the first pregnancy, and BMI, PCOS, ART in the second pregnancy (determined by clinical significance and *P* value less than 0.1 in univariate analysis). In the multivariate analysis, Variance Inflation Factor (VIF) between age of first pregnancy, IPI, and age of second pregnancy was calculated to determine whether all three factors should be included in the regression model. In addition, IPI was included as a continuous variable in the regression model to obtain the predicted risk of GDM in the second pregnancy. The linear regression analyzed the correlation between IPI months and predicted risk of GDM and the significance of the difference between slopes was tested using covariance analysis. *P* < 0.05 indicated a statistically significant difference.

## Results

**Fig. 1:**
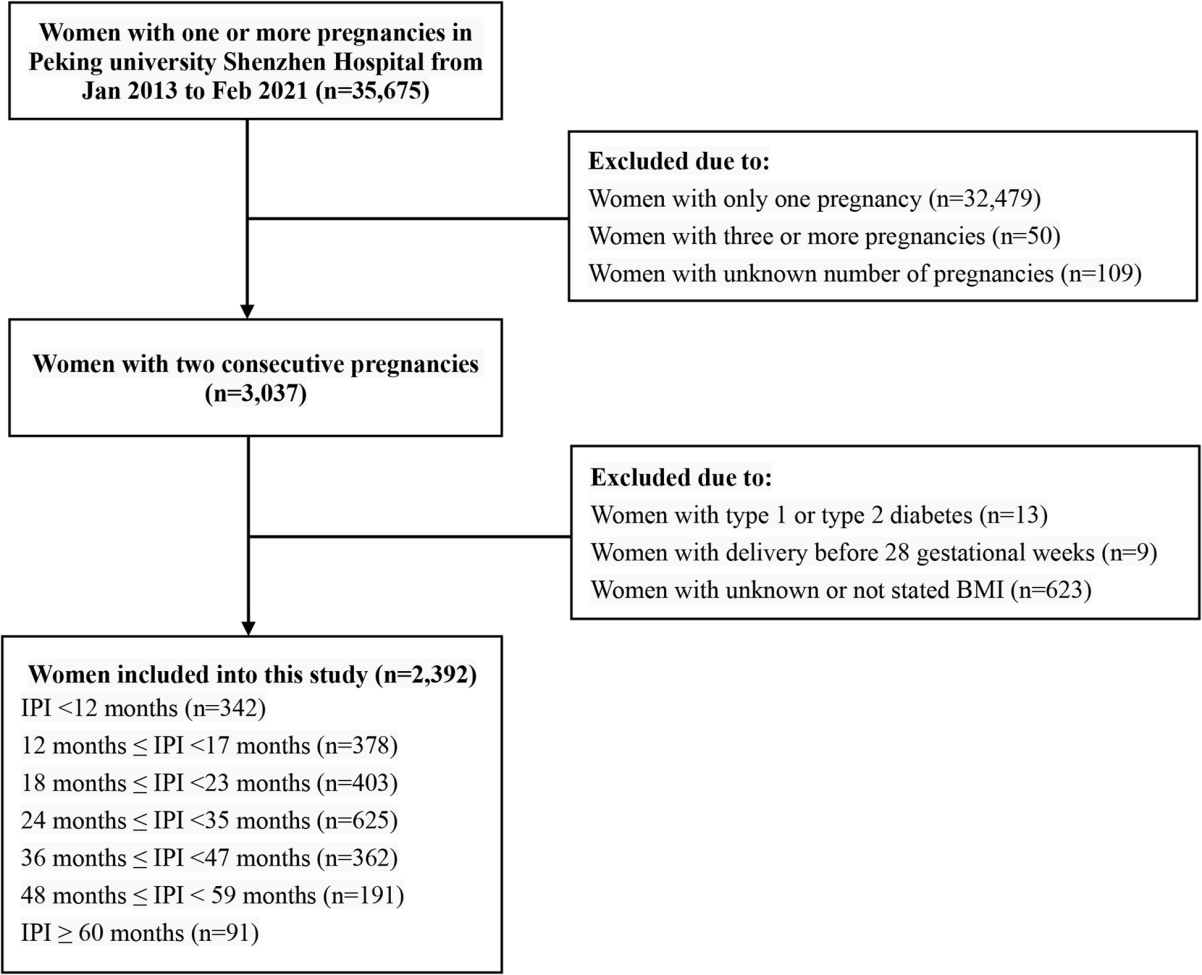
Flow chart showing inclusion and exclusion in this study. IPI: interpregnancy interval; BMI: body mass index

 A total of 35,675 female participants with at least one pregnancy in Peking University Shenzhen Hospital were recorded from January 2013 to February 2021. After 33,283 were disqualified according to the exclusion criteria, a total of 2,392 participants with two consecutive single deliveries were included (Fig. 1).

The overall median IPI was 24.72 months (IQR: 15.61–36.60 months). The median IPI of the GDM group was significantly longer than that of the non-GDM group (*P* < 0.05). The maternal age; BMI in the first pregnancy; proportion of cases with GDM, hypertensive diseases, and cesarean section in the first pregnancy; and BMI of the second pregnancy in the GDM group were all significantly greater than those in the non-GDM group (*P* < 0.05; Table 1).

**Table 1:** Univariate analysis of risk factors for the GDM in the second pregnancy

Risk factors		Total (*n* = 2392)	GDM group (*n* = 306)	Non-GDM group (*n* = 2086)	t/Z /χ²	*P*
IPI [months (interquartile range)]		24.72(15.61–36.60)	29.10(18.38-41.00)	23.94(15.26–35.75)	4.700	<0.001^a^
Maternal age in the first pregnancy (years, x ± s)		28.27 ± 3.35	29.3 ± 3.54	28.12 ± 3.29	5.826	0.000
Parity in the first pregnancy (n, x ± s)		1.08 ± 0.29	1.09 ± 0.32	1.08 ± 0.29	0.850	0.396
Year of delivery in the first pregnancy						
2016–2021 [n (%)]		971(40.59)	117(38.24)	854(40.94)	0.809	0.368
Before 2016 [n (%)]		1421(59.41)	189(61.76)	1232(59.06)		
Occupation in the first pregnancy						
Employed [n (%)]		2168(90.64)	27(8.82)	1894(90.80)	0.494	0.482
Unemployed [n (%)]		224(9.36)	3(0.98)	192(9.20)		
Medical payment method						
With insurance [n (%)]		1924(80.43)	256(83.66)	1668(79.96)	2.319	0.128
Without insurance [n (%)]		468(19.57)	50(16.34)	418(20.04)		
Nationality						
Ethnic Han [n (%)]		2275(95.11)	291(95.10)	1984(95.11)	<0.001	0.993
Minority [n (%)]		117(4.89)	15(4.90)	102(4.89)		
Marital status in the first pregnancy						
Married [n (%)]		2358(98.58)	299(97.71)	2059(98.71)	1.237	0.266
Unmarried or divorce[n/%]		34(1.42)	7(2.29)	27(1.29)		
Sex of newborn in the first pregnancy						
Male [n (%)]		1194(49.92)	151(49.35)	1043(50.00)	0.046	0.831
Female [n (%)]		1198(50.08)	155(50.65)	1043(50.00)		
Birth weight (g, x ± s)		3225.29 ± 449.96	3248.77 ± 491.56	3221.85 ± 443.56	0.906	0.366
BMI of the first pregnancy (kg/m^2^, x ± s)		20.50 ± 2.66	21.31 ± 3.01	20.38 ± 2.58	5.160	<0.001
BMI of the second pregnancy (kg/m^2^, x ± s)		21.29 ± 2.98	22.32 ± 3.30	21.14 ± 2.90	5.931	<0.001
GDM in the first pregnancy						
Yes [n (%)]		270(11.29)	127(41.50)	143(6.86)	319.923	<0.001
No [n (%)]		2122(88.71)	179(58.50)	1943(93.14)		
TD in the first pregnancy						
Yes [n (%)]		156(6.52)	17(5.56)	139(6.66)	0.537	0.464
No [n (%)]		2236(93.48)	289(94.44)	1947(93.34)		
HDCP in the first pregnancy						
Yes [n (%)]		83(3.47)	21(6.86)	62(2.97)	12.059	0.001
No [n (%)]		2309(96.53)	285(93.14)	2024(97.03)		
PTB in the first pregnancy						
Yes [n(%)]		146(6.10)	26(8.50)	120(5.75)	3.506	0.061
No [n (%)]		2246(93.90)	280(91.50)	1966(94.25)		
CS in the first pregnancy						
Yes [n (%)]		754(31.52)	127(41.50)	627(30.06)	16.196	<0.001
No [n (%)]		1638(68.48)	179(58.50)	1459(69.94)		

^a^tested by Mann-Whitney *U*-test; *IPI *Interpregnancy interval, *BMI *Body mass index, *GDM *Gestational diabetes mellitus, *TD *Thyroid disease, *HDCP *Hypertensive disorder complicating pregnancy, *PTB *Preterm birth, *CS *Cesarean section

Out of the total cases of GDM in the first pregnancy, which amounted to 270, 20.4% (55 out of 270) were found to be overweight or obese. Furthermore, among the 220 individuals who were overweight or obese during their first pregnancy, 25.0% (55 out of 220) were diagnosed with GDM.

After being sorted by the median IPI of 24 months, the proportion of participants who were overweight or obese with a short IPI (< 24 months) was found to be 9.35%. This percentage was not significantly different from the proportion of overweight or obese participants with a long IPI (≥ 24 months), which was 9.06% (*χ*²=0.059, *P* = 0.808).

Furthermore, the difference in BMI between the first and second pregnancy was calculated for both groups. In participants with a short IPI (< 24 months), the difference in BMI was found to be 0.72 ± 2.10 kg/m^2^. This difference was not significantly different from the difference in BMI observed in participants with a long IPI (≥ 24 months), which was 0.86 ± 1.89 kg/m^2^ (*t* = 1.742, *P* = 0.082). In participants with GDM in the first pregnancy, the difference in BMI (0.68 ± 2.09 kg/m^2^) was not significantly different from that in participants without GDM in the first pregnancy, (0.81 ± 1.98 kg/m^2^)(*t* = 0.937, *P* = 0.349).

There was a significant collinear effect between the age of the first pregnancy and the age of the second pregnancy (VIF = 55.08 and 47.93), so we did not include the age of the second pregnancy in the analysis to avoid the interaction effects. After adjustment for potential confounding factors (maternal age; proportion of cases with PCOS, ART, GDM, HDCP, preterm birth, and cesarean section in the first pregnancy; proportion of cases with PCOS, ART in the second pregnancy; and BMI in the first and second pregnancy) in the multivariate logistic regression, longer IPI categories (24–36 months, 36–48 months, 48–60 months, and IPI ≥ 60 months) were independently associated with increased risk of GDM in the second pregnancy (*P* < 0.05), and the adjusted odds ratio values increased with the IPI (Table 2). However, overall multivariate analysis showed no significant difference in GDM risk in the second pregnancy existed between the participants with a shorter IPI (< 12 months or 12–18 months) and those with the reference IPI category (18–24 months;*P* > 0.05). When we examined the cut-off value for the first pregnancy at 35 years old, we found that the subgroup of advanced pregnancies had an insufficient sample size (only 88 cases). Therefore, we modified the cut-off value to 30 years old, which resulted in both subgroups having a sufficient sample size. Compared to the reference IPI, an IPI of 36 months or more was significantly associated with an increased risk of GDM in the second pregnancy, regardless of whether the maternal age during the first pregnancy was older than 30 years (*P* < 0.05; Table 3). However, when participants were younger than 30 years of age, an IPI of 60 months or more was not significantly associated with GDM risk. The small number of GDM cases in this group (only 3 GDM cases) may have influenced the insignificant results. Conversely, an IPI of less than 36 months was not found to be significantly associated with GDM risk (Table 3).

**Table 2:** Multivariate analysis of the risk factors for GDM in the second pregnancy

Variables	Regression coefficient	Standard error	Wald value	*P*	OR	95%CI for OR
IPI < 12 months	-0.001	0.278	0.000	0.997	0.999	0.580–1.721
12 months ≤ IPI < 18 months	0.155	0.259	0.360	0.549	1.168	0.703–1.940
18 months ≤ IPI < 24 months	reference					
24 months ≤ IPI < 36 months	0.461	0.225	4.210	**0.040**	**1.585**	**1.021–2.462**
36 months ≤ IPI < 48 months	0.868	0.240	13.105	**<0.001**	**2.381**	**1.489–3.809**
48 months ≤ IPI < 60 months	0.912	0.279	10.687	**0.001**	**2.488**	**1.441–4.298**
IPI ≥ 60 months	0.942	0.349	7.278	**0.007**	**2.565**	**1.294–5.087**
Maternal age in the first pregnancy	0.059	0.021	7.696	**0.006**	**1.060**	**1.017–1.105**
BMI in the first trimeter of the first pregnancy	-0.023	0.036	0.419	0.517	0.977	0.911–1.048
PCOS in the first pregnancy	-0.067	1.109	0.004	0.952	0.936	0.106–8.226
ART in the first pregnancy	0.012	0.319	0.001	0.971	1.012	0.541–1.892
GDM in the first pregnancy	2.190	0.154	202.037	**<0.001**	**8.933**	**6.605–12.082**
HDCP in the first pregnancy	0.466	0.314	2.196	0.138	1.594	0.860–2.952
PTB in the first pregnancy	0.285	0.260	1.202	0.273	1.330	0.799–2.216
CS in the first pregnancy	0.099	0.148	0.447	0.504	1.104	0.826–1.474
BMI in the first trimeter of the second pregnancy	0.087	0.031	7.944	**0.005**	**1.091**	**1.027–1.160**
PCOS in the second pregnancy	-0.249	1.468	0.029	0.865	0.780	0.044–13.838
ART in the second pregnancy	-0.111	0.447	0.061	0.805	0.895	0.373–2.151

*IPI *Interpregnancy interval, *BMI *Body mass index, *PCOS *Polycystic ovary syndrome, *ART *Assisted reproductive technology, *GDM *Gestational diabetes mellitus, *HDCP *Hypertensive disorder complicating pregnancy, *PTB *Preterm birth, *CS *Cesarean section

**Table 3:** Subgroup analysis of the risk of GDM in the second pregnancy

		Adjusted OR and 95%CI					
	IPI < 12 months	12 months ≤ IPI < 18 months	18 months ≤ IPI < 24 months	24 months ≤ IPI < 36 months	36 months ≤ IPI < 48 months	48 months ≤ IPI < 60 months	IPI ≥ 60 months
Maternal age < 30 years (1596 cases)	0.636(0.289–1.398)	0.893(0.457–1.744)	1.000	1.372(0.778–2.419)	**2.250(1.254–4.037)**	**2.053(1.014–4.159)**	1.849(0.742–4.606)
Maternal age ≥ 30 years (796 cases)	1.477(0.660–3.305)	1.574(0.711–3.483)	1.000	1.898(0.944–3.818)	**2.387(1.076–5.293)**	**3.245(1.363–7.727)**	**3.729(1.298–10.718)**
BMI < 24 kg/m^2^(2172 cases)	1.074(0.592–1.948)	1.319(0.758–2.295)	1.000	**1.760(1.085–2.855)**	**2.515(1.498–4.224)**	**2.843(1.575–5.135)**	2.184(0.987–4.834)
BMI ≥ 24 kg/m^2^ (220 cases)	0.636(0.148–2.724)	0.542(0.120–2.450)	1.000	0.732(0.216–2.480)	2.855(0.807–10.103)	0.777(0.141–4.299)	3.910(0.818–18.679)
Without GDM (2122 cases)	0.670(0.339–1.324)	0.818(0.438–1.528)	1.000	1.177(0.704–1.970)	**1.880(1.105-3.200)**	**2.121(1.167–3.852)**	1.961(0.917–4.194)
Combined with GDM (270 cases)	2.321(0.894–6.025)	**2.619(1.074–6.386)**	1.000	**3.747(1.652–8.499)**	**4.356(1.724–11.005)**	2.879(0.865–9.581)	**5.373(1.078–26.793)**

*IPI *Interpregnancy interval, *BMI *Body mass index, *GDM *Gestational diabetes mellitus

Furthermore, we compared the GDM risk associated with IPI in the subgroup of participants with the first pregnancy age of less than 30 years to that in the subgroup with the first pregnancy age of 30 years or older. We found that when the IPI was greater than or equal to 36 months, there was no significant difference in GDM risk between the two subgroups (*P* < 0.05). Conversely, when the IPI was in the range of 24–36 months or less than 18 months, the risk of GDM in the subgroup with maternal age of 30 years or older was significantly higher than that in the subgroup with maternal age less than 30 years (*P* < 0.05) (Supplementary Table 1). Similarly, when we compared the GDM risk associated with the same IPI in the subgroup of participants younger than 30 years of age during the second pregnancy to the subgroup with participants aged 30 years or older, the results were consistent with that of comparison based on the age group during the first pregnancy (Supplementary Table 2).

Among participants with a BMI below 24 kg/m^2^ in the first trimester, the IPIs at 24–36 months, 36–48 months, and 48–60 months showed a significant positive association with the risk of GDM (*P* < 0.05). However, in participants with a BMI below 24 kg/m^2^, we did not observe a significant association between any IPI category and the risk of GDM (*P* > 0.05).

Specifically, among participants who had GDM in their first pregnancy, those with an IPI of 12–18 months or an IPI of 24 months or longer had a significantly higher risk of GDM compared to the reference IPI (*P* < 0.05). On the other hand, among participants who did not have GDM in their first pregnancy, the minimum IPI that was significantly associated with an increased risk of GDM was 36–48 months (Table 3).

**Fig. 2:**
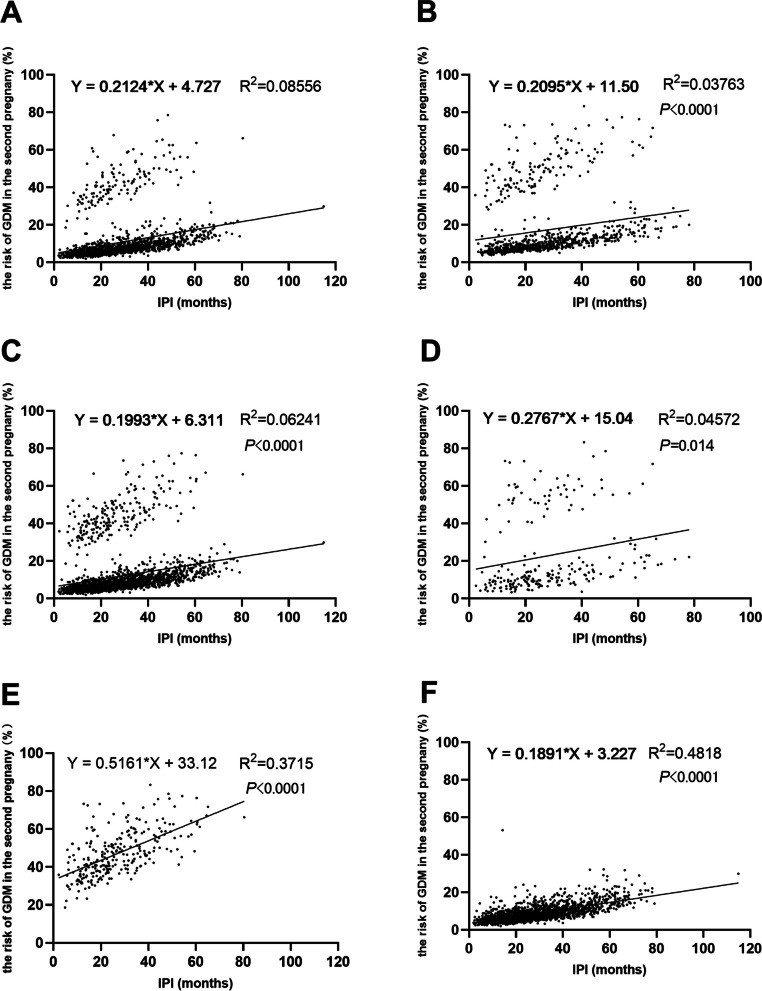
Linear regression results of IPI on the risk of GDM in the second pregnancy in stratified analyses Legend: **A** Performed on participants aged less than 30 years in their first pregnancy; **B **Performed on participants aged 30 years or older in their first pregnancy; **C** Performed on participants with a BMI less than 24 kg/m2 in their first pregnancy; **D** Performed on participants with a BMI of 24 kg/m2 or higher in their first pregnancy; **E** Performed on participants with GDM in their first pregnancy; **F** Performed on participants without GDM in their first pregnancy. IPI: interpregnancy interval; GDM: gestational diabetes mellitus

 The results of linear regression revealed a positive correlation between IPI (months) and GDM predicted risk across all participants (*P* < 0.01). When participants were divided into groups based on their history of GDM, the R-squared value significantly increased to 0.3715 and 0.4818 (Fig. 2). Among women with a history of GDM, the slope value of linear regression was significantly higher (0.5161) compared to women without a history of GDM (0.1891) (*F* = 284.168, *P* = 0.000).

## Discussion

Our study suggests that a long IPI may be associated with an increased risk of GDM in the second pregnancy. As shown in Table 2, for women with an IPI of 24 months or greater, the adjusted odds ratio value of GDM risk increased from 1.585 to 2.565 when compared with the reference IPI (18–24 months). For those with an IPI of less than 24 months, no significant differences existed in the GDM risk in the second pregnancy. Therefore, within the range of IPI recommended by the WHO [12], the risk of GDM in the second pregnancy is lowest when the IPI is 18–24 weeks. For women planning a second pregnancy, our findings will provide useful information to select an appropriate IPI.

Some previous reports have also supported the association between a long IPI and increased GDM risk in the second pregnancy. In Holmes et al.’s study [20], the interval (2.9 years) between the first and second pregnancies with recurrent GDM was significantly higher than that (2.4 years) without recurrent GDM. Khambalia et al. [21] also reported that a long interval from the previous pregnancy was a risk factor for recurrent GDM. The study of Hanley et al. [14] showed that a long IPI was associated with a significant increase in GDM risk using unmatched (between-participant) analyses, whereas this association disappeared in the matched analyses. A nationwide data analysis from the United States found that a long IPI (36 months or greater) was associated with an increased risk of GDM [15]. However, that study included a very high proportion of participants with obesity (mean BMI greater than 26 kg/m^2^) and the odds ratio value was not corrected for GDM in previous pregnancies. Women with GDM in the previous pregnancy are at high risk of recurrent GDM in the second pregnancy [5], and this should be adjusted as a confounding factor.

The results of this study indicates that the link between a prolonged IPI and the risk of GDM is not a result of maternal age. Both stratified multivariate analysis (Table 3) and subgroup comparison analysis (Supplemented Table 1) demonstrate that the risk of GDM is significantly higher when the IPI is equal to or greater than 36 months, regardless of whether the mother is less than 30 years old or 30 years old or older during her first pregnancy. Additionally, the results from stratified analysis based on the age of the second pregnancy align with those from stratified analysis based the age of the first pregnancy (Supplementary Table 2).

This study suggests that if GDM is present during the first pregnancy, the risk of developing GDM in the second pregnancy is not only associated with a long interpregnancy interval (IPI), but also with a short IPI (12–18 months). A short IPI may prevent the body from fully recovering from the effects of GDM in the previous pregnancy, leading to a higher recurrence rate in the second pregnancy. Major et al. [22] suggested that an IPI of 24 months or less was also the most significant risk factor for a recurrence of GDM. In a nationwide data analysis from the United States, a short IPI (6–17 months) was also associated with an increased risk of GDM [15]. Hanley et al.‘s study [14] also supported that a short IPI is linked to an increased risk of GDM. However, Hanley et al.‘s study [14] did not consider whether the first pregnancy included GDM and suggested that a short IPI is related to insufficient time for weight loss after pregnancy, resulting in an increased risk of GDM in the second pregnancy. Nevertheless, in this study, the percentage of women who needed to lose weight was relatively small (less than 10% of women who were overweight or obese in their first trimester), and their weight loss attempts were minimal or non-existent (BMI change of less than 1 unit). Furthermore, there was no significant change in BMI between their two pregnancies, regardless of whether the first pregnancy was associated with GDM or if the IPI exceeded 24 months. Therefore, it is believed that the relationship between IPI and the risk of GDM in the second pregnancy was not influenced by weight loss. In contrast to women with no history of GDM, women with a history of GDM were more likely to be affected by IPI, regardless of changes in BMI. Women with a history of GDM may need a narrower range for their IPI (18–24 weeks) in order to reduce their risk of GDM. For women without a history of GDM, their physiological recovery may be faster, making a short IPI irrelevant to the risk of GDM in the second pregnancy. However, if the IPI is too long (e.g., 36 months or more in this study), the advantages of the physiological adaptations that occurred in the previous pregnancy are no longer available, resulting in an increased risk of GDM.

Our results are inconsistent with the study of Gebremedhin et al. [23], in which the minimum IPI that significantly increased the risk of GDM during the second pregnancy was 48 months and 24 months for participants with and without GDM during the first pregnancy, respectively. In our study, women with deliveries before 28 weeks were excluded, whereas those delivering after 20 weeks were enrolled in the study of Gebremedhin et al. [23] The differences between the two studies may be related to differences in demographic characteristics.

However, Gebremedhin et al. [16] found that an IPI of less than 18 months had a protective effect against GDM. A large-sample study from the United States suggested that intervals of less than 6, 6–11, and 12–17 months had a significant overall protective effect against GDM (aRR: 0.89–0.98) [24]. These studies indicate that the association between a short IPI and GDM remains unclear. According to the recommendations of ACOG [25] and the WHO [12], the pregnancy interval should be at least 18 to 24 months because a short IPI increases the risk of preterm birth, spontaneous prematurity, smallness for gestational age, and other adverse pregnancy outcomes [26]. Therefore, a short IPI is not recommended even if it is a protective factor for GDM.

The mechanism by which a prolonged IPI increases the risk of GDM is still unknown. Some studies have speculated that this association is related to a gradual decline in maternal physiological functions [27] (such as increased uterine blood flow and other physiological and anatomical adaptations of the reproductive system) after childbirth. In case of a long period after childbirth without another pregnancy, the mother’s physiological functions may become similar to those of a first-time mother. Another hypothesis is that unobserved metabolic or anatomic factors may contribute to delayed fertility and poor delivery outcomes [28]. It has been suggested that 18 to 23 months is the optimal IPI for preventing adverse perinatal outcomes [28].

Our study has some limitations. First, since this was a retrospective single-center study, the inclusion of cases may be biased, and some participant information may not have been collected comprehensively, such as whether an abortion before 28 weeks occurred between the two included pregnancies. Second, some confounding factors were not considered in the analysis, such as the family history of diabetes, hyperlipidemia, smoking, diet, and exercise habits. Third, because this was an unmatched study, confounding factors are inevitable. Fourth, the small sample size of obese or older women may have biased the findings.

## Conclusion

This study conducted at a single center in China has shown that a longer interpregnancy interval (IPI) is linked to a higher likelihood of developing gestational diabetes mellitus (GDM) in subsequent pregnancies. Importantly, this risk is not influenced by the mother’s age. Moreover, for women who have previously had GDM, having a shorter IPI also increases the risk of GDM recurrence. These findings can be utilized by women to make informed decisions about the appropriate length of time between pregnancies in order to minimize the risk of GDM in subsequent pregnancies.

### Supplementary Information

Supplementary Material 1.

Supplementary Material 2.

## Data Availability

The datasets used and/or analysed during the current study available from the corresponding author on reasonable request.
