# Supplementary material for: Effect of interpregnancy interval on the risk of gestational diabetes mellitus during a second pregnancy
Source: BMC Pregnancy Childbirth. 2024 Jun 4;24:406. doi: 10.1186/s12884-024-06602-z (PMC11151626; doi:10.1186/s12884-024-06602-z)
Supplement: Supplementary file 1 — Supplementary Material 1. [file 12884_2024_6602_MOESM1_ESM.docx]

Supplementary Table 1 Comparison of the GDM risk between the two subgroups based on the maternal age of the first pregnancy

|  | Maternal age ＜30 years in the first pregnancy | |  | Maternal age ≥30 years in the first pregnancy | | *χ*² | *P* |
| --- | --- | --- | --- | --- | --- | --- | --- |
|  | N | GDM [n(%)] |  | N | GDM [n(%)] |  |  |
| IPI <12 months | 223 | 11(4.93) |  | 119 | 18(15.13) | **10.389** | **0.001** |
| 12 months ≤ IPI <18 months | 263 | 20(7.60) |  | 115 | 21(18.26) | **9.396** | **0.002** |
| 18 months ≤ IPI <24 months | 259 | 23(8.88) |  | 144 | 16(11.1) | 0.527 | 0.468 |
| 24 months ≤ IPI <36 months | 414 | 47(11.35) |  | 211 | 37(17.54) | **4.593** | **0.032** |
| 36 months ≤ IPI <48 months | 258 | 43(16.67) |  | 104 | 21(20.19) | 0.633 | 0.426 |
| 48 months ≤ IPI <60 months | 120 | 19(15.83) |  | 71 | 14(19.72) | 0.471 | 0.492 |
| IPI ≥ 60 months | 59 | 8(13.56) |  | 32 | 8(25.00) | 1.874 | 0.171 |
