# Supplementary material for: Effect of interpregnancy interval on the risk of gestational diabetes mellitus during a second pregnancy
Source: BMC Pregnancy Childbirth. 2024 Jun 4;24:406. doi: 10.1186/s12884-024-06602-z (PMC11151626; doi:10.1186/s12884-024-06602-z)
Supplement: Supplementary file 2 — Supplementary Material 2. [file 12884_2024_6602_MOESM2_ESM.docx]

Supplementary Table 2 Comparison of the GDM risk between the two subgroups based on the maternal age of the second pregnancy

|  | Maternal age ＜30 years in the second pregnancy | |  | Maternal age ≥30 years in the second pregnancy | | *χ*² | *P* |
| --- | --- | --- | --- | --- | --- | --- | --- |
|  | N | GDM [n(%)] |  | N | GDM [n(%)] |  |  |
| IPI <12 months | 182 | 8(4.40) |  | 160 | 21(13.13) | **8.361** | **0.004** |
| 12 months ≤ IPI <18 months | 172 | 9(5.23) |  | 206 | 32(15.53) | **10.287** | **0.001** |
| 18 months ≤ IPI <24 months | 156 | 14(8.97) |  | 247 | 25(10.12) | 0.144 | 0.704 |
| 24 months ≤ IPI <36 months | 149 | 10(6.71) |  | 476 | 74(15.55) | **7.614** | **0.006** |
| 36 months ≤ IPI <48 months | 61 | 13(21.31) |  | 301 | 51(16.94) | 0.665 | 0.415 |
| 48 months ≤ IPI <60 months | 17 | 3(17.65) |  | 174 | 30(17.24) | 0.000 | 1.000 |
| IPI ≥ 60 months | 4 | 0(0.00) |  | 87 | 16(18.39) | N/A | 1.000^*^ |

^*^by Fisher’s test
